# The complete mitochondrial genome of *Hypseleotris cyprinoides* (Perciformes: eleotridae)

**DOI:** 10.1080/23802359.2023.2197085

**Published:** 2023-04-10

**Authors:** Zhongchao Pan, Xinhe Ruan, Huitao Cheng, Chunli Zhang, Huihong Zhao

**Affiliations:** a Guangdong Weilai Biotechnology Co. Ltd, Guangzhou, China; b College of Marine Sciences, South China Agricultural University, Guangzhou, China

**Keywords:** Mitochondrial genome, *Hypseleotris cyprinoides*, phylogenetic analysis

## Abstract

In this study, we obtained the complete mitochondrial genome of *Hypseleotris cyprinoides*, which was 16520 bp in length. The mitogenome contained 37 genes, including the typical set of 13 protein-coding genes (PCGs), 22 transfer RNA (tRNA) genes, and 2 Ribosomal RNA (rRNA) genes. A, C, G, and T distribution was 28.57%, 29.91%, 16.99%, and 24.53%, respectively. The length of the total protein-coding genes was 11441 bp, which accounts for 66.80% of the whole mitochondrial genome. The Maximum Likelihood (ML) phylogenetic analysis based on the concatenated nucleotide sequences of 13 PCGs showed that *H.cyprinoides* as a sister species to *Hypseleotris klunzingeri* was clustered in the family Hypseleotris. The discovery of the complete mitochondrial genome of *H.cyprinoides* would help to conduct in-depth research on Hypseleotris.

## 1. Introduction

**Figure 1.**
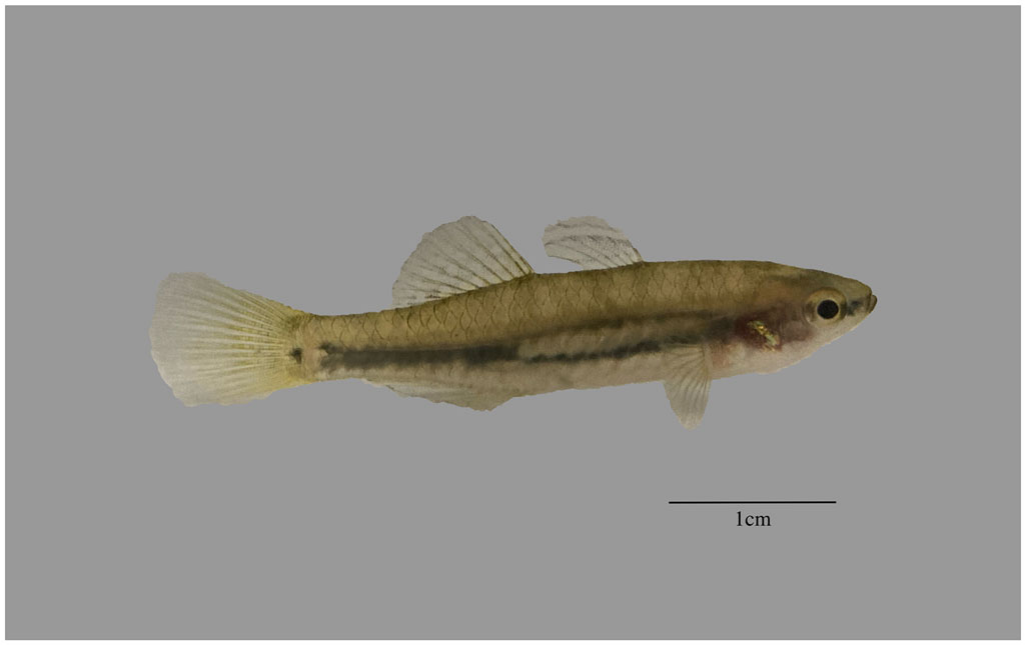
Image of *Hypseleotris cyprinoides. H. cyprinoides* has a head and body that is strongly flattened from side to side, and a small mouth that does not reach the front edge of the eye socket. The image token by author Xinhe Ruan in Guangdong China.

*Hypseleotris cyprinoides* (Valenciennes, 1837) belongs to the genus of *Hypseleotris* of Eleotridae family, Gobioidei of Perciformes, which distributes in the estuary region of China, Japan, Southeast Asian countries in Southwest Pacific. *H.cyprinoides* have a strongly laterally compressed head and body, a small mouth not reaching the anterior border of the orbit, an elongated body cavity with several anal pterygiophores preceding the first vertebral hemal spine, and an ovoid blotch at the dorsal base of the pectoral fin (Figure 1, This image was taken by author Xinhe Ruan). Although it is classified into the Eleotridae by morphological method, there is almost no analysis of the gene sequence of *H.cyprinoides* (Thacker and Unmack 2005). The Mitochondrial genome has been widely used for phylogenetic studies in recent years, and several new perspectives other than traditional morphological classification have been proposed (Miya et al. 2003). Therefore, to clarify its evolutionary status at the level of molecular, we determined the mitochondrial genome sequence of *H. cyprinoides* and analyzed its evolutionary characteristics, which will help us to improve the data at the molecular level and clarify the phylogenetic relationship and taxonomic status of *H. cyprinoides* in this study.

## 2. Materials and methods

### 2.1. Sample collection and preservation

The specimen of *Hypseleotris cyprinoides* was obtained from Pingtung City, Taiwan Province, China (N22°0854′, E120°7474′) in November 2021. This specimen is deposited in the Laboratory of Aquatic Economic Animal Germplasm Resources and breeding Engineering, South China Agricultural University, China (Xinhe Ruan, rxh.equal@outlook.com), under voucher number CHT2150001.

### 2.2. DNA extraction and sequencing and phylogenetic analysis method

Total genomic DNA was extracted using a modified cetyltrimethylammonium bromide (CTAB) method and applied to 500-bp paired-end library construction using the NEBNext Ultra DNA Library Prep Kit for Illumina sequencing. Sequencing was carried out on the Illumina NovaSeq 6000 platform (BIOZERON Co., Ltd., Shanghai, China), and using a run configuration of 2 × 150 bp to generate approximately 5 Gb of data for each sample. Eventually, clean Data was spliced using SPAdes v3.14.1 software, the assembled sequence was reordered and oriented according to the reference mitochondrial genome (Zhang et al. 2000), thus generating the final assembled mitochondrial genomic sequence. The mitogenome was assembled from 6934 Mb raw reads, with mean depth of 400×. The GC content obtained therein was 40.63%. The mitochondrion genes were annotated using the online MITOS tool, using default parameters to predict protein-coding genes, transfer RNA (tRNA) genes, and ribosome RNA (rRNA) genes. The base composition was calculated and the phylogenetic tree was built using MEGA X software (Kumar et al. 2018).

## 3. Results and discussion

### 3.1. Characteristics of H. cyprinoides mitochondrial genome

**Figure 2.**
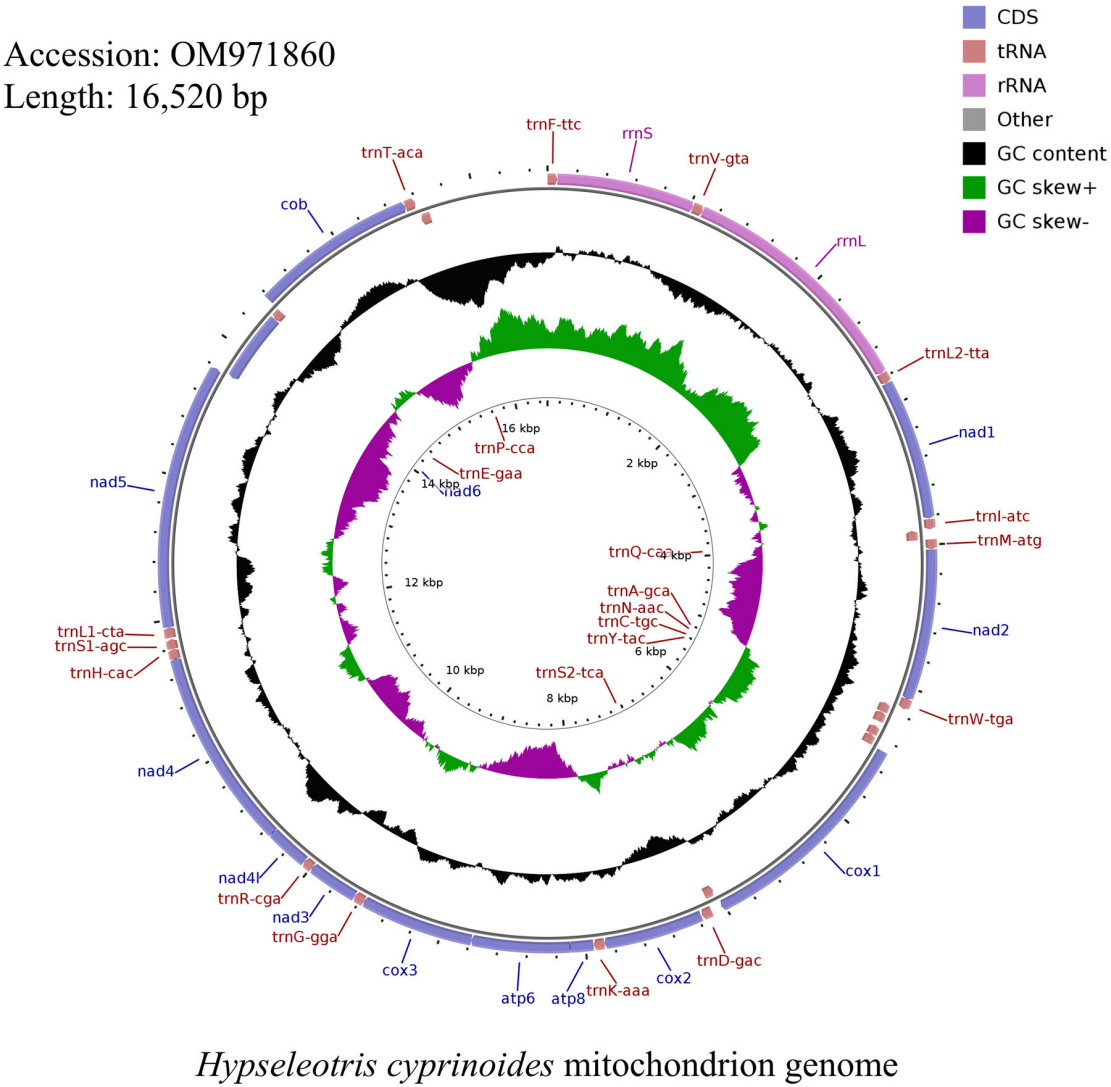
Image and mitochondrion genome map of *Hypseleotris cyprinoides*. CDS: Coding sequence; tRNA: Transfer RNA; rRNA: Ribosomal RNA.

The complete mitogenome of *H. cyprinoides* was 16,520 bp in length (GenBank accession number: OM971860) and contained the typical set of 13 protein-coding genes (PCGs), 22 transfer RNA (tRNA) genes, 2 Ribosomal RNA (rRNA) genes (Table 1). And the software CGView was used to map the mitochondrial genome (Figure 2). Its composition is similar to that of typical vertebrates (Miya et al. 2001). The distribution of A, C, G, and T was 28.57%, 29.91%, 16.99%, and 24.53% respectively. The length of the total protein-coding genes was 11,441 bp, which accounts for 66.80% of the whole mitochondrial genome, and the base composition was 25.96% for A, 31.19% for C, 16.51% for G, and 26.34% for T. Most of the mitochondrial genes were encoded in the H chain, except for *ND6* and 8 tRNA genes (*Gln*, *Ala*, *Asn*, *Cys*, *Tyr*, *Ser*, *Glu*, and *Pro*), which were encoded by the L chain. Most PCGs began with a start codon ATG except the *COX1* gene, which initiated with GTG. This is similar to the mitochondrial DNA of the Eleotridae family (Zang et al. 2016; Meng et al. 2016). Ten PCGs terminated with a complete stop codon TAA or TAG, whereas the other three, including the *COX2*, *NAD4*, and *COB* genes, ended with others.

**Table 1.** The organization of the complete mitochondrial genome in *Hypseleotris cyprinoides.*

Gene	Strand	Position	Size(bp)	Intergenic spacer	Start coden	Stop coden
*tRNA-Phe*	F	1–68	68	–	–	–
*rrnS*	F	69–1020	952	0	–	–
*tRNA-Val*	F	1021–1092	72	0	–	–
*rrnL*	F	1094–2772	1679	1	–	–
*tRNA-Leu2*	F	2773–2847	75	0	–	–
*nad1*	F	2848–3822	975	0	ATG	TAG
*tRNA-Ile*	F	3827–3896	70	4	–	–
*tRNA-Gln*	R	3896–3966	71	−1	–	–
*tRNA-Met*	F	3966–4034	69	−1	–	–
*nad2*	F	4035–5081	1047	0	ATG	TAG
*tRNA-Trp*	F	5080–5151	72	−2	–	–
*tRNA-Ala*	R	5156–5224	69	4	–	–
*tRNA-Asn*	R	5226–5298	73	1	–	–
*tRNA-Cys*	R	5336–5401	66	37	–	–
*tRNA-Tyr*	R	5402–5472	71	0	–	–
*cox1*	F	5474–7027	1554	1	GTG	TAA
*tRNA-Ser2*	R	7028–7098	71	0	–	–
*tRNA-Asp*	F	7102–7173	72	3	–	–
*cox2*	F	7180–7870	691	6	ATG	T
*tRNA-Lys*	F	7871–7944	74	0	–	–
*atp8*	F	7946–8113	168	1	ATG	TAA
*atp6*	F	8104–8787	684	−10	ATG	TAA
*cox3*	F	8787–9572	786	−1	ATG	TAA
*tRNA-Gly*	F	9572–9643	72	−1	–	–
*nad3*	F	9644–9994	351	0	ATG	TAG
*tRNA-Arg*	F	9993–10061	69	−2	–	–
*nad4l*	F	10062–10358	297	0	ATG	TAA
*nad4*	F	10352–11737	1386	−7	ATG	AGA
*tRNA-His*	F	11733–11801	69	−5	–	–
*tRNA-Ser1*	F	11802–11869	68	0	–	–
*tRNA-Leu1*	F	11880–11952	73	10	–	–
*nad5*	F	11953–13791	1839	0	ATG	TAA
*nad6*	R	13788–14309	522	−4	ATG	TAA
*tRNA-Glu*	R	14310–14378	69	0	–	–
*cob*	F	14383–15523	1141	4	ATG	T
*tRNA-Thr*	F	15524–15595	72	0	–	–
*tRNA-Pro*	R	15596–15665	70	0	–	–

In the column intergenic length, the positive number indicates interval base pairs between genes, while the negative number indicates the overlapping base pairs between genes.

### 3.2. Phylogenetic analysis

**Figure 3.**
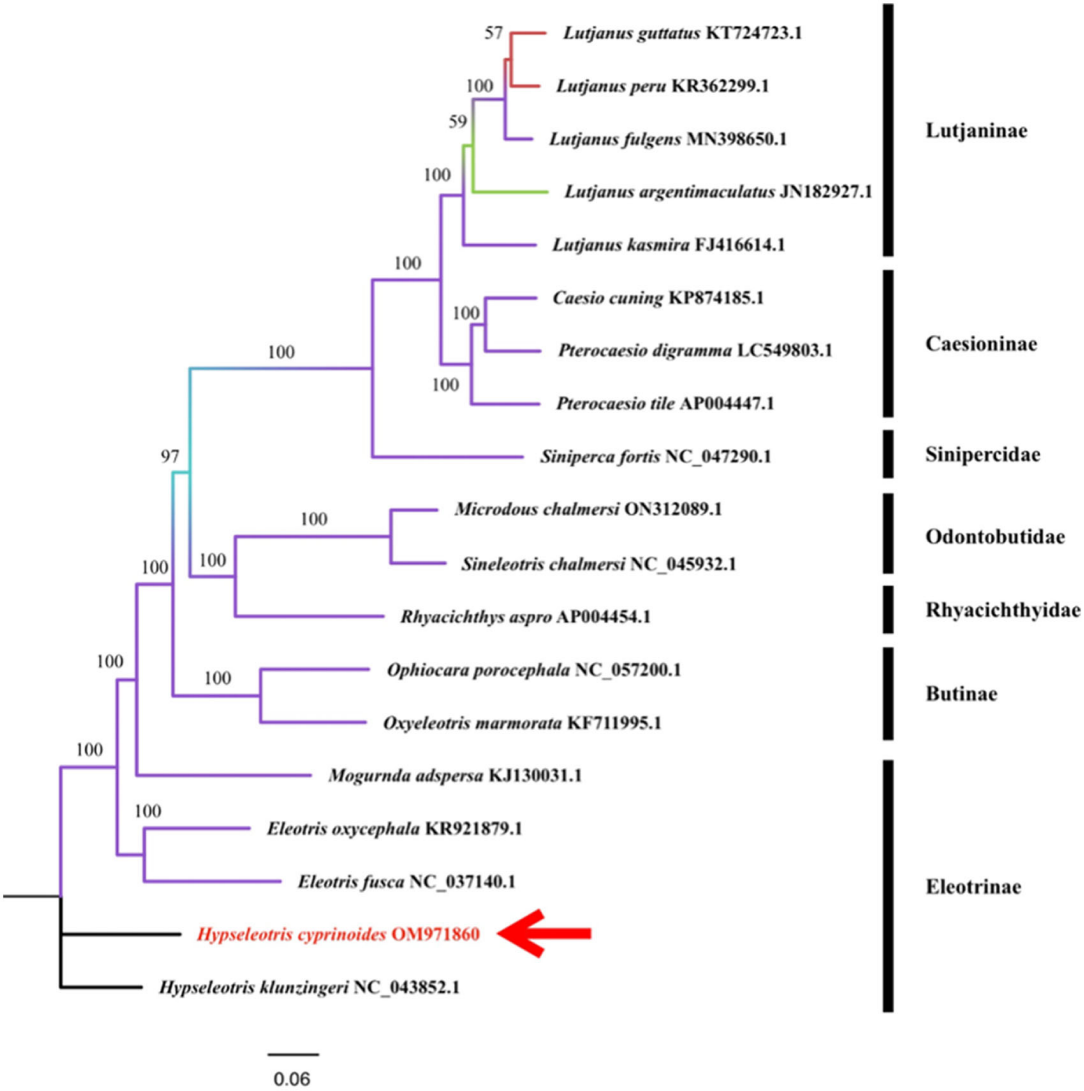
A phylogenetic tree for *H. cyprinoides* and other 18 species based on assembled nucleotide sequences of 13 protein-coding genes, two rRNA genes, and 22 tRNA genes. The base composition was calculated and the phylogenetic tree was built using MEGA X software. The number on each node indicates the values of the ultrafast bootstrap (UFB) of 1000 replications. The phylogenetic position of *H. cyprinoides* was marked with a red arrow. The following sequences were used: *Eleotris oxycephala* KR921879.1 (Meng et al. 2016), *Eleotris fusca* NC_037140.1 (unpublished), *Hypseleotris klunzingeri* NC 043852.1 (Schmidt et al. 2019b), *Mogurnda adspersa* KJ130031.1 (Perini et al. 2016), *Ophiocara porocephala* NC_057200.1 (unpublished), *Oxyeleotris marmorata* KF711995.1 (Xu et al. 2016), *Microdous chalmersi* ON312089.1 (Wang et al. 2019), *Sineleotris chalmersi* NC 045932.1 (Wang et al. 2019), *Rhyacichthys aspro* AP004454.1 (Miya et al. 2003), *Lutjanus guttatus* KT724723.1 (Bayona-Vásquez et al. 2017), *Lutjanus peru* KR362299.1 (Bayona-Vásquez et al. 2017), *Lutjanus fulgens* MN398650.1 (Afriyie et al. 2020), *Lutjanus argentimaculatus* JN182927.1 (unpublished), *Lutjanus kasmira* FJ416614.1 (unpublished), *Casio cuing* KP874185.1 (Zhan et al. 2017), *Pterocaesio digramma* LC549803.1 (Song et al. 2020), *Pterocaesio tile* AP004447.1 (Miya et al. 2003), *Siniperca fortis* NC 047290.1(Peng et al. 2020).

The Maximum Likelihood (ML) phylogenetic tree was built based on 13 PCG of 19 species’ complete mitochondrial genomes. All the mitochondrial gene sequences were downloaded from the NCBI gene bank. The number at each node has been obtained from the probability by 1000 bootstrap. From the phylogenetic tree (Figure 3), we can see that the genome of *H. cyprinoides* is most similar to that of *Hypseleotris klunzingeri* among the species of Hypseleotris analyzed. This is consistent with the results of morphological classification. Both *H. cyprinoides* and *Hypseleotris klunzingeri* belong to the genus of Hypseleotris. According to Australian researchers (Schmidt and McDougall 2019), the *Hypseleotris klunzingeri* is a freshwater estuary fish endemic to Australia, which is comparable to where we collected the specimen. It is speculated that the separation of the Earth’s plates resulted in geographical isolation and the emergence of two comparable species. It will provide better phylogenetic insights into this species.

## 4. Conclusions

We reported the first complete mitochondrial genome assembly and annotation of *H. cyprinoides* using next-generation sequencing technology. The circular mitogenome was 16,520 bp in length, contained 37genes encoding 13 PCGs, 22 tRNAs, and two rRNAs. The phylogenetic tree was inferred by a Maximum-likelihood phylogenetic tree based on the sequences of 18 species, which supported that *H. cyprinoides* was grouped together with *Hypseleotris klunzingeri*. The mitochondrial genomic data of *H. cyprinoides* provided in this study will aid future research on the evolution, taxonomy, DNA barcoding, and population genetics of Hypseleotris species.

## Data Availability

The genome sequence data that support the findings of this study are openly available in GenBank of NCBI at https://www.ncbi.nlm.nih.gov/ under accession no. OM971860. The associated Bio-Project, SRA, and Bio-Sample numbers are PRJNA858347, SRR20183523, and SAMN29706239 respectively.
